# High geomagnetic field intensity recorded by anorthosite xenoliths requires a strongly powered late Mesoproterozoic geodynamo

**DOI:** 10.1073/pnas.2202875119

**Published:** 2022-07-11

**Authors:** Yiming Zhang, Nicholas L. Swanson-Hysell, Margaret S. Avery, Roger R. Fu

**Affiliations:** ^a^Department of Earth and Planetary Science, University of California, Berkeley, CA 94720;; ^b^US Geological Survey, Geology, Minerals, Energy, and Geophysics Science Center, Moffett Field, CA 94035;; ^c^Department of Earth and Planetary Sciences, Harvard University, Cambridge, MA 02138

**Keywords:** absolute paleointensity, anorthosite, geodynamo, Proterozoic, inner core

## Abstract

Obtaining estimates of Earth’s magnetic field strength in deep time is complicated by nonideal rock magnetic behavior in many igneous rocks. In this study, we target anorthosite xenoliths that cooled and acquired their magnetization within ca. 1,092 Ma shallowly emplaced diabase intrusions of the North American Midcontinent Rift. In contrast to the diabase which fails to provide reliable paleointensity estimates, the anorthosite xenoliths are unusually high-fidelity recorders yielding high-quality, single-slope paleointensity results that are consistent at specimen and site levels. An average value of ∼83 ZAm^2^ for the virtual dipole moment from the anorthosite xenoliths, with the highest site-level values up to ∼129 ZAm^2^, is higher than that of the dipole component of Earth’s magnetic field today and rivals the highest values in the paleointensity database. Such high intensities recorded by the anorthosite xenoliths require the existence of a strongly powered geodynamo at the time. Together with previous paleointensity data from other Midcontinent Rift rocks, these results indicate that a dynamo with strong power sources persisted for more than 14 My ca. 1.1 Ga. These data are inconsistent with there being a progressive monotonic decay of Earth’s dynamo strength through the Proterozoic Eon and could challenge the hypothesis of a young inner core. The multiple observed paleointensity transitions from weak to strong in the Paleozoic and the Proterozoic present challenges in identifying the onset of inner core nucleation based on paleointensity records alone.

## Significance

Acquiring high-fidelity ancient magnetic field intensity records from rocks is crucial for constraining the long-term evolution of Earth’s core. However, robust estimates of ancient field strengths are often difficult to recover due to alteration or nonideal behavior. We use rocks known as anorthosite that formed in the deep crust and were brought to the near surface where they acquired thermal remanent magnetizations. These rocks have experienced minimal postformation alteration and yield high-quality paleointensity estimates. In contrast to scenarios of a progressively decaying field leading up to a proposed late nucleation of Earth’s inner core, these data record a strong field 1.1 Ga. A strong field that persisted over a 14-My interval indicates the existence of appreciable power sources for Earth’s dynamo at this time.

Earth’s magnetic field is the result of convective flow of liquid iron alloy in Earth’s outer core. At present day, the geodynamo is collectively powered by heat flow across the core–mantle boundary and from the crystallization of the solid inner core from the liquid outer core which provides latent heat and compositional buoyancy due to the exclusion of light elements (1). However, while paleomagnetic studies have found that a dynamo field has existed since at least 3.4 Ga (2–5), Earth’s inner core likely crystallized more recently. Estimates of the timing of the initial crystallization of Earth’s inner core are interconnected with estimates for the core’s thermal conductivity. Higher conductivity values imply faster cooling rates to maintain the geomagnetic field, which in turn imply that the threshold for the crystallization of the inner core happened more recently (6). While some estimated thermal conductivity values are consistent with an inner core age >3 Ga (7, 8), other estimates have implied higher thermal conductivity values and an age for the inner core that is less than 1.5 Ga (9–13), with some suggesting even younger ages (<700 Ma) (14–16). The possibility of late inner core nucleation has motivated proposals of novel power sources to sustain the geomagnetic field through early Earth history including precipitation of light element minerals such as MgO (17–19) and SiO_2_ (20) at the core–mantle boundary. Given that estimates for the core’s thermal conductivity continue to be debated, it is crucial to use observational records as an independent constraint on the thermal evolution of Earth’s core and mantle.

Paleomagnetic records from ancient rocks are one of the few types of observational data that have the potential to provide constraints on the thermal evolution of Earth’s core. Evidence for a persistent magnetic field through the Proterozoic, for example, likely necessitates the existence of plate tectonics that sustained core–mantle boundary heat flow (21). However, strikingly low estimates of geomagnetic field strengths have been obtained ca. 565 Ma during the Ediacaran Period (22–24) and ca. 370 Ma during the Devonian Period (25–27), potentially indicating unusual periods of core dynamo activity at those times. The Ediacaran data have been interpreted to indicate that there was a progressively decaying field up to that time that was soon followed by initial crystallization of the inner core (22). Sparse paleointensity data in the Proterozoic Era (2,500 to 539 Ma) and the reality of high variability in Phanerozoic Era records (539 to 0 Ma) necessitate additional data to evaluate interpreted trends.

**Fig. 1.**
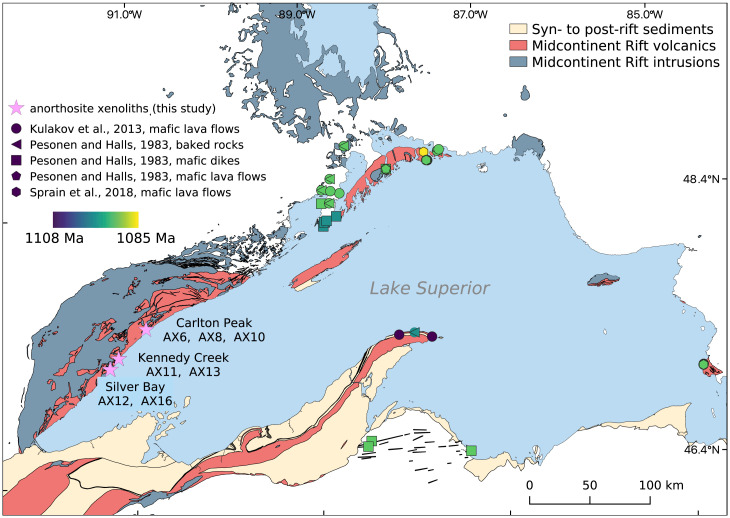
Simplified geologic map of the Lake Superior region showing the distribution of rocks associated with the late Mesoproterozoic Midcontinent Rift. Purple stars mark sites with paleointensity results that passed the selection criteria from this study. Paleomagnetic sites from ref. 29 are categorized by lithology. All sites from refs. 29–31 are color-coded by their ages.

Determinations of the absolute value of ancient geomagnetic field strength rely on igneous rocks that acquire thermal remanent magnetizations (TRMs) as they cool. These magnetizations need to be unmodified by subsequent heating or chemical alteration in order to maintain the record of the ancient geomagnetic field from the time of cooling. Intracontinental magmatic events are an important target for determination of ancient paleointensity as they can be well preserved within continental interiors. This interior position results in them typically being distant from tectonic events along continental margins that can drive alteration through heat and fluid flow. However, intraplate magmatism associated with large igneous provinces is typically of geologically short duration with the bulk of magmatic products emplaced within 1 My or less (28). The Midcontinent Rift (Fig. 1) is an exception as it is a large igneous province where magmatism lasted ∼25 My from ca. 1,109 to 1,084 Ma during which there were pulsed intervals of more rapid magmatic activity (32). Additionally, extension ceased in the Midcontinent Rift prior to lithospheric separation, preserving volcanic, intrusive, and sedimentary rocks of the rift within the continental interior far from the continental margin and subsequent orogenesis. As a result, rocks of the rift have unusually simple paleomagnetic behavior for their greater than 1 Ga age, and paleomagnetic data from rift rocks form a central record of Mesoproterozoic paleogeography (21). The duration of magmatic activity within the Midcontinent Rift is longer than the entire 20.4-My-long Neogene Period such that it provides an extended well-preserved window into the intensity of Earth’s magnetic field in the late Mesoproterozoic.

Despite the excellent preservation of the rocks, nonideal paleointensity behaviors have posed challenges for the interpretation of many previous paleointensity results from the Midcontinent Rift (29–31). The most trusted type of paleointensity estimate is that obtained through experiments in which the primary natural remanent magnetization (NRM) is progressively replaced by a laboratory magnetization that is imparted in a known field with internal consistency checks (such as in IZZI-style Thellier experiments) (33). In such Thellier paleointensity experiments, one typical departure from ideal behavior due to the presence of nonuniformly magnetized grains [with either multidomain (34) or vortex states (35)] is sagging or double-slopes as visualized in Arai plots that show TRM acquired versus NRM lost. For such data, distinct paleointensity estimates may be calculated depending on the interpreter’s choice of slope. Typically, such nonideal behavior would result in a higher paleointensity estimate from the steeper-sloped low-temperature portion of the experiment and a lower paleointensity estimate from the high-temperature portion. For example, in data from the Midcontinent Rift, ref. 29 used the low-temperature slope as the best representation of the past magnetic field strength (likely overestimating the field strength), whereas ref. 30 used the high-temperature slope (likely underestimating the field strength). Such nonideal results were rejected by ref. 31, who applied stricter paleointensity selection criteria, but as a result had few accepted sites.

In this study, we target the high-purity anorthosite xenoliths of the Beaver River diabase in the Midcontinent Rift. While magmatic activity within the Midcontinent Rift was protracted, there were intervals of particularly rapid volcanism and voluminous emplacement of intrusions (32). The ca. 1,092 Ma Beaver Bay Complex in northern Minnesota punctuates one such period of magmatism during the main stage of Midcontinent Rift activity. The magma that formed the 1,091.7 ± 0.2 Ma Beaver River diabase dikes and sills of the Beaver Bay Complex transported numerous anorthosite xenoliths that have short-axis diameters up to 180 m via wide conduits (36, 37). These anorthosite xenoliths are plagioclase cumulates that formed comagmatically with the host diabase in the lower crust—an interpretation confirmed by U-Pb zircon geochronology (38). They are attractive targets for paleomagnetic study as plagioclase crystals can protect magnetic inclusions from alteration. In addition, the alteration of the plagioclase crystals does not readily result in the formation of secondary iron oxides in contrast with Fe-silicate minerals such as olivine and pyroxene. The anorthosite xenoliths targeted in this study were brought to the near surface in magma that formed hypabyssal (shallowly emplaced) intrusions of the Beaver River diabase (38). They would have been heated to tholeiitic magma temperature (∼1,100 ^∘^C) which is below the melting point of the xenolith plagioclase (70% anorthite), but well above the Curie temperature of magnetite (38). Fe-Ti oxides within the plagioclase likely exsolved above magnetite Curie temperature (39) and subsequently cooled and acquired TRMs in conjunction with the host diabase at a paleolatitude of 22 ± 2^∘^ (calculated from the paleomagnetic pole of the coeval Portage Lake Volcanics) (40). Paleointensity experiments on the anorthosite xenoliths have a high success rate, yielding consistent specimen- and site-level paleointensity results. The anorthosite xenoliths show low anisotropy of TRM acquisition and can acquire TRM linearly within the range of relevant field strengths. Magnetic imaging shows that the anorthosite specimens have dominant magnetic carriers within and interstitial to plagioclase crystals without strong preferred orientations. Thermal modeling results and paleomagnetic directional data show that the anorthosite xenoliths acquired TRMs while cooling with the Beaver River diabase (38). Stepwise thermal demagnetization data show the anorthosite xenoliths to have dominantly single-component magnetizations that often unblock sharply within temperature ranges between 500 and 580 ^∘^C, consistent with remanence being held by low-titanium titanomagnetite (38).

## Results and Interpretations

### Petrography and Magnetic Imaging of Anorthosite Xenoliths.

**Fig. 2.**
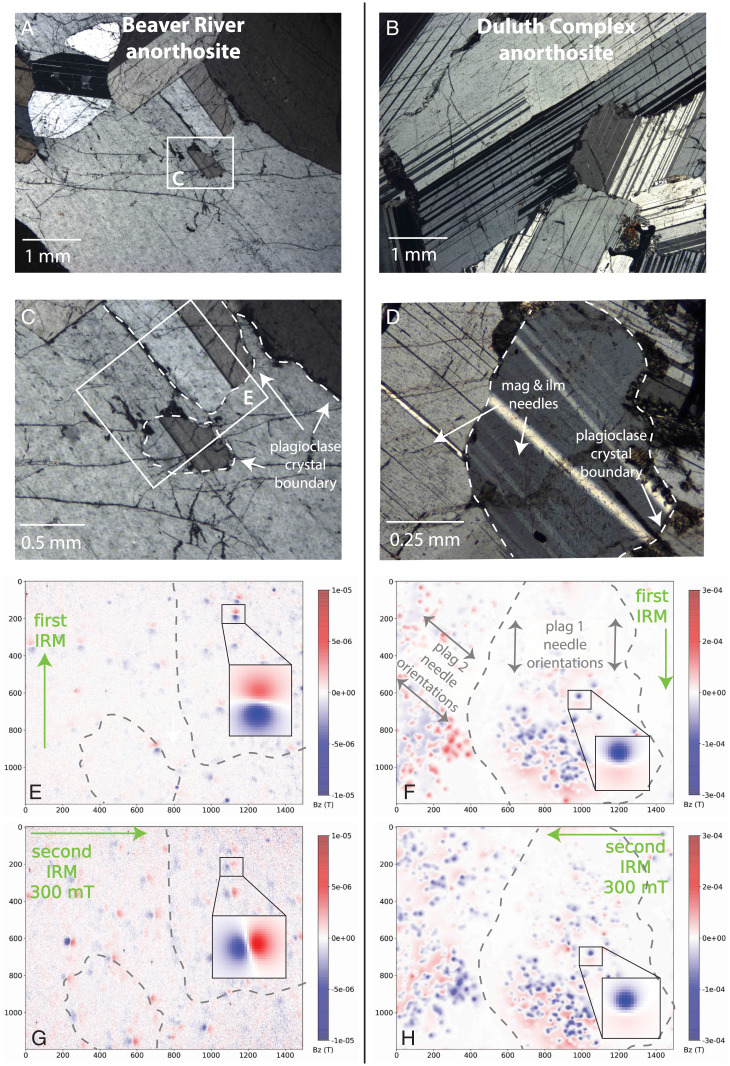
(*A*–*D*) Thin section petrographic images and (*E*–*H*) magnetic maps of an anorthosite sample from the Beaver River anorthosite xenolith in the Silver Bay region from which paleomagnetic site AX16 and geochronology sample MS99033 were collected (*A*, *C*, *E*, and *G*) (38) and from a distinct anorthosite within the Duluth Complex Anorthositic Series (*B*, *D*, *F*, and *H*). The Duluth Complex anorthosites were not targeted for paleointensity experiments in this study given complexities associated with more pronounced fabrics. Cross-polarized petrographic images of the Beaver River anorthosite (*A* and *C*) reveal plagioclase with a granoblastic texture of crystals that are largely free of large opaque inclusions. In contrast, plagioclase crystals in the Duluth Complex anorthosite have euhedral, interlocking crystals with an igneous foliation (*B*), and the plagioclase crystals contain abundant Fe-Ti oxide needles that have preferred orientations that are often parallel with the [001] axis of the plagioclase. Maps of the vertical component of magnetic field (B*_z_*) developed with a QDM show relatively weak magnetic sources within plagioclase crystals in the Beaver River anorthosite (in *E*) relative to the strongly magnetic large oxide needles within Duluth Complex plagioclase (in *F*). The B*_z_* color scale is an order of magnitude greater in the Duluth Complex anorthosite maps (*F* and *H*) than the maps for the Beaver River anorthosite (*E* and *G*). *E* and *G* and *F* and *H* show experiments performed on both samples where we apply a first field along the *y* axis of the field of view and then apply a second field of 300 mT orthogonal to the first field direction. The remanent magnetizations acquired by both anorthosites were mapped after the application of each field. The magnetic images show that remanent magnetizations of Beaver River anorthosite (i.e., the individual dipoles visible with paired red +B*_z_* and blue –B*_z_* lobes) align well with the first applied field direction (*E*) and then rotated to align with the second applied field direction indicating minimal anisotropic behavior (*G*). In contrast, the remanent magnetizations carried by the magnetic needles in plagioclase 1 of the Duluth anorthosite xenolith align well with the first applied field, but those in plagioclase 2 acquired an oblique remanence direction with respect to the field direction (*F*). After the application of an orthogonal 300 mT external field, magnetization of those needles in plagioclase 1 did not change direction due to strong shape anisotropy, whereas the magnetization of needles in plagioclase 2 flipped but with the acquired remanence remaining oblique to the field direction (*H*). Insets in *E*–*H* show example dipole directions (fit using the algorithm of ref. 41) in response to the application of orthogonal IRMs. The directional changes of the Beaver River anorthosite xenolith sources indicate a lower magnetic anisotropy compared to the relative lack of change for the Duluth Complex anorthosite xenolith sources. The unit of the axes in the QDM maps are in µm.

The dominantly monomineralic anorthosite xenoliths within the Beaver River diabase often have granoblastic texture characterized by equigranular crystals with weakly developed petrofabrics (Fig. 2*A*). Plagioclase crystals within coarse-grained intrusions often contain abundant elongate Fe-Ti oxide inclusions that are visible with optical microscopy (42–44). The Beaver River anorthosite xenoliths lack such large (tens of µm) oxide inclusions such that the oxides are not visible within the plagioclase crystals using optical microscopy (Fig. 2*C*). Magnetic imaging using a quantum diamond microscope (QDM), however, shows that there are magnetic remanence carriers within the plagioclase crystals (Fig. 2 *E* and *G*). The sources of these magnetic remanence carriers are likely to be Fe-Ti oxides that formed within plagioclase crystals above the Curie temperature of magnetite (38, 39). To highlight these distinctive aspects of the Beaver River anorthosite xenoliths, we present petrographic and magnetic imaging data from the Beaver River anorthosite as well as an anorthosite sample from the Duluth Complex Anorthositic Series—an older intrusive complex within the Midcontinent Rift that was not targeted for paleointensity experiments in this study (Fig. 2). In contrast to the Beaver River anorthosite, the plagioclase crystals of this Duluth Complex anorthosite sample have a pronounced igneous foliation (Fig. 2*B*). In addition, there are abundant Fe-Ti oxide needles within the Duluth Complex plagioclase grains that are typically aligned with the [001] axes of the crystals (Fig. 2*D*). Magnetic imaging confirms that these needles have magnetic moments oriented along their long axes (Fig. 2 *F* and *H*). As a result of this shape anisotropy, remanent magnetizations are acquired at angles highly oblique to applied fields (Fig. 2*F*). In an experiment where a second orthogonal field was applied, the first isothermal remanent magnetizations (IRM) of a set of magnetite needles within a plagioclase grain either fail to rotate or flip by 180^∘^. When they flip, they remain in a direction that is oblique to the applied field direction dictated by shape anisotropy (Fig. 2 *F* and *H*). These experiments enable visualization of the grain-scale magnetic anisotropy of elongated exsolved (titano)magnetite inclusions within plagioclase grains that leads to magnetic anisotropy observed at the bulk sample scale (42, 45). In contrast, the remanent magnetizations of the ferromagnetic grains imaged in the Beaver River anorthosite xenoliths, targeted for paleointensity in this study, align with the applied field directions indicating minimal remanence anisotropy (Fig. 2 *E* and *G*). The relative lack of petrofabrics and minimal grain-scale magnetic anisotropy make the Beaver River anorthosite xenoliths a particularly compelling target for paleointensity experiments.

### Paleointensity.

**Fig. 3.**
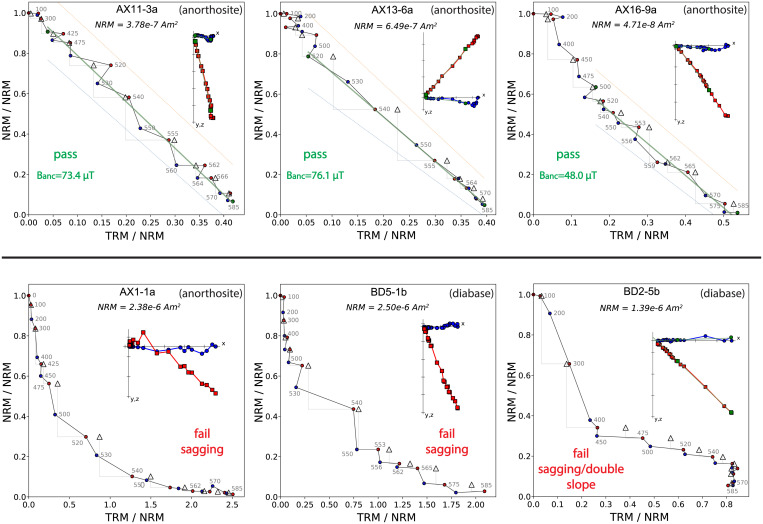
Example results of paleointensity experiments are displayed on Arai plots, and zero-field heating results are shown as orthogonal plots (Zijderveld plots; *Insets*) for anorthosite and diabase specimens. Green squares and lines in Arai plots and green squares in orthogonal plots show the range of data points used for fitting. Red (blue) circles indicate zero-field/in-field (in-field/zero-field) steps (ZI [IZ]). Triangles mark pTRM checks. Blue and red squares in the Zijderveld plots are X–Y and X–Z projections, respectively, of the NRMs in specimen coordinates. Dashed red and blue lines show bounding regions associated with the SCAT statistic (46). (*Top*) Successful specimen paleointensity results with straight, single-slope behaviors that pass our selection criteria. The green lines represent fits for the dominant single-slope component that passes the acceptance criteria and gives an estimate of the ancient field strength (B*_anc_*). (*Bottom*) Plots for anorthosite specimens AX1-1a and diabase BD5-1b show nonideal sagging behavior that fails our acceptance criteria. Specimen BD2-5a is an example where the data appear linear with distinct slopes in the low and high temperature ranges such that it could pass less restrictive selection criteria, particularly if a narrower temperature range was used for the experiment. Data analysis and visualization was conducted using PmagPy (47).

**Fig. 4.**
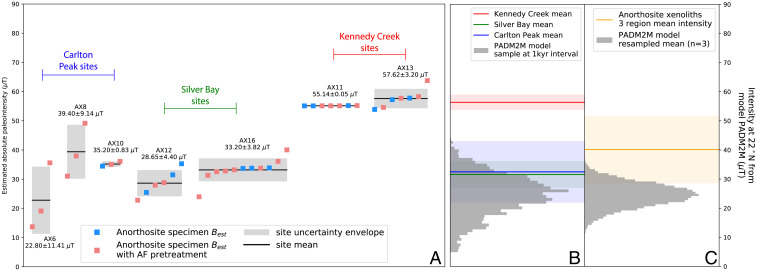
(*A*) Summary plot of individual specimen absolute paleointensity results (square symbols) and their averages and SDs at site level (black bars with gray 1 SD uncertainty envelopes) where each AX site is an individual anorthosite xenolith within the Beaver River diabase. All results are corrected for cooling rate bias with a factor of 0.75. The sites with successful experiments come from three regions (Carlton Peak, Silver Bay, and Kennedy Creek) which would have cooled at distinct times yielding similar estimates within each region with differences between regions. (*B*) Regional means calculated from the specimen-level data are compared to the distribution of intensities calculated from a PADM2M (4 8) at the latitude corresponding to the paleolatitude of study region (22^∘^N). (*C*) The mean of the three regional means is compared to means calculated from three random values drawn from the PADM2M model (48). The distribution represents a total of 10,000 iterations of taking three random draws and calculating the mean. These comparisons highlight that these anorthosites’ paleointensity values are strong in relation to the geomagnetic field over the past 2 My. All shaded regions in *B* and *C* represent 1 SD uncertainties.

Following IZZI-style paleointensity experiments (33), 40 from a total of 86 anorthosite specimens and 0 out of a total of 69 diabase specimens passed our paleointensity result selection criteria (*Materials and Methods*). Seven anorthosite sites and no diabase sites have specimen results that pass these selection criteria. Example NRM/TRM (Arai) plots are shown in Fig. 3. Summary specimen absolute paleointensity estimates and site-level mean paleointensity values are plotted in Fig. 4 (and provided in *SI Appendix*, Table S1) where each site represents an individual anorthosite xenolith. The paleointensity quality indexes (Q*_PI_*) (49) for the anorthosite xenolith sites are all 5 or 6 (*SI Appendix*, Table S2). The cooling rate-corrected absolute paleointensity estimates from the anorthosite xenoliths have a mean of 38.86 ± 12.10 µT. The site mean virtual dipole moment is ∼83 ZAm^2^ (10^21^ Am^2^) ca. 1,092 Ma. All measurement-level paleointensity experiment data are available within the MagIC database https://earthref.org/MagIC/doi/10.1073/PNAS.2202875119.

Typical paleointensity experimental data of the anorthosite specimens have straight, single-slope NRM/TRM plots, and the accepted fractions of temperature steps span over the origin-trending, primary remanence components (Fig. 3). We accept specimen- and site-level absolute paleointensity results from those anorthosite xenoliths that pass the selection criteria. Other anorthosite xenoliths and diabase specimens failed the selection criteria largely because of double-slope or sagging behavior (fail FRAC selection; *Materials and Methods*), poor partial TRM (pTRM) checks, and zigzagging behaviors occasionally superimposed on top of sagging behavior (fail SCAT and deviation angle [DANG] selection; *Materials and Methods* and Fig. 3). A 20-mT alternating field (AF) treatment after in-field heating steps was applied to some specimens, but this treatment did not result in significant changes in the experimental results for the anorthosite xenolith or diabase specimens (Fig. 4).

In addition to estimating paleointensity values by introducing a set of selection criteria to filter our experiment results, we applied an independent statistical method from ref. 50 to all experimental data regardless of their NRM/TRM plot statistics to make a bias-corrected estimation of paleointensity. This Bayesian probabilistic method is based on the assumption that paleointensity estimates from specimens that come from a same cooling unit are distributed around a true paleointensity value with the various deflections being expressed as the curvature parameter of the NRM/TRM plot (51). The posterior paleointensity distributions from these sites with high-quality specimen-level data are in agreement with the site-level averages developed using the selection criteria approach (Fig. 4 and *SI Appendix*, Fig. S1). Overall, the high-quality results from the anorthosite xenoliths of the Beaver River diabase indicate that the anorthosite xenoliths record a high geomagnetic field ca. 1,092 Ma.

### Rock Magnetism.

#### Coercivity.

**Fig. 5.**
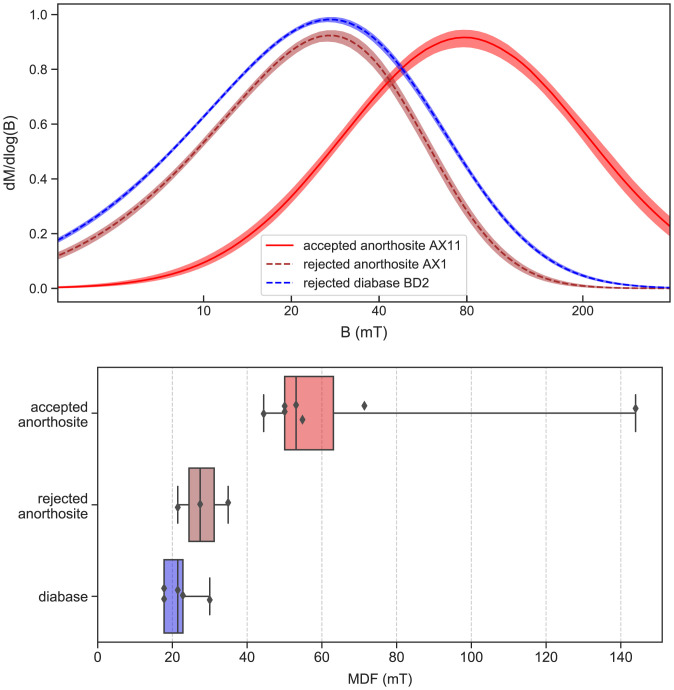
(*Top*) Example coercivity spectra of anorthosite and diabase rock chips from sites that pass or fail our paleointensity selection criteria. (*Bottom*) Box plots of MDF values associated with back-field demagnetization for all anorthosite and diabase specimens with single-component coercivity unmixing results. Bars in the boxes show median values, and the whiskers show the minimum and the maximum value of the datasets. Both plots show that anorthosite specimens that pass paleointensity selection criteria have higher coercivities consistent with a higher portion of single-domain–like magnetite grains than the other anorthosite specimens and the diabase.

Additional rock magnetic data indicate that those anorthosite specimens which pass the paleointensity selection criteria contain dominant magnetic remanence carriers with magnetic properties similar to stoichiometric, noninteracting, single-domain magnetite, whereas anorthosite samples that failed the paleointensity result selection along with all diabase samples have more pronounced populations of nonideal carriers. Magnetic property measurement system (MPMS) data show that both the diabase and anorthosite contain (titano)magnetite as evidenced by the presence of the Verwey transition (*SI Appendix*, Fig. S2) (52, 53). Anorthosite specimens from sites that yield successful paleointensity results have Verwey transition temperatures near 120 K as expected for stoichiometric magnetite with minimal Ti content (54). However, diabase and anorthosite specimens that did not pass our paleointensity selection typically have Verwey transitions that are suppressed toward lower temperatures (*SI Appendix*, Fig. S2), indicating that their magnetic grains either have relatively higher Ti content or have been partially oxidized (54). Another difference is that samples which pass paleointensity selection have distinctly higher average median destructive field (MDF) values than other anorthosite and diabase specimens (Fig. 5). Single-component fits for coercivity spectra (55) show that anorthosites having successful paleointensity results can contain magnetic grain populations that have higher coercivities. For these successful samples, MDF values associated with back-field demagnetization experiments range from 44 to 144 mT with a median of 53 mT (Fig. 5). In contrast, other anorthosite and diabase specimens tend to have lower peak coercivities (MDF range of 18 to 35 mT with a median of 22 mT; Fig. 5). This result is consistent with an interpretation that magnetic grain populations with more multidomain-like behavior are responsible for the nonideal paleointensity behaviors during experiments of such specimens (56).

#### Bulk TRM anisotropy.

Significant remanence anisotropy has been documented to exist within certain anorthositic rocks that formed in layered intrusive complexes (42, 45). Strong remanence anisotropy associated with the igneous foliation developed within anorthosite from the Stillwater Complex has been shown to cause significant overestimation or underestimation of paleointensity values depending on the relative orientations between the fabrics and an applied magnetic field (45). To assess whether our paleointensity estimates are biased by bulk remanence anisotropy, we calculated the gamma statistic, which is the angular difference between the last pTRM step of paleointensity experiment and the applied field direction. The results show that the anorthosite specimens used in our paleointensity experiment have low gamma values ranging from 0.9^∘^ to 11.9^∘^, with a median value of 4.2^∘^ (*SI Appendix*, Table S1). Because these anorthosite specimens were oriented at various directions with respect to the outcrops, the angles between the applied laboratory field direction during paleointensity experiments with respect to any fabrics within the anorthosite specimens are expected to cover a wide range of angles. These gamma values of the anorthosite xenolith bulk specimens are similar to those of the Midcontinent Rift volcanics studied by ref. 30. Therefore, the bulk Beaver River anorthosite xenoliths do not have significant remanence anisotropy. Paleodirectional data from our anorthosite xenoliths indicate that they have minimal remanence anisotropy as their site mean directions closely match those of the Beaver River diabase hosts without deviating due to a fabric (38). These bulk results are consistent with the data from the grain-scale magnetic anisotropy experiments that also show minimal remanence anisotropy (Fig. 2 *E* and *G*).

### Considering Secular Variation and Cooling Rate.

To best characterize the geomagnetic axial dipole field intensity during a certain time period, a paleointensity dataset should cover a sufficient amount of time such that paleosecular variations of the geomagnetic field are averaged. Thermal modeling results from ref. 38 indicate that the Beaver River anorthosite xenoliths were heated to tholeiitic magma temperature (∼1,100 ^∘^C)—lower than the melting temperature of the anorthosite plagioclase (given its composition of 70% anorthite) (57, 58)—and acquired TRM during cooling with their diabase host on a time scale of a few thousand years, partially averaging secular variation within single sites. Another consequence of the anorthosite xenoliths having slowly cooled in the interior of thick diabase intrusions is that slow cooling rates can bias paleointensity estimates toward higher values (59). Large differences in cooling rates between acquisition of an NRM in nature versus a TRM in the laboratory can result in overestimated paleointensities for single-domain grains (59–61). From the thermal history model of ref. 38, we can estimate the duration over which the diabase and anorthosite cooled from the Curie temperature of magnetite (∼580 ^∘^C) to the time when they blocked in the majority of their characteristic natural remanence magnetization (∼500 ^∘^C; Fig. 3) (38). We find the cooling time to ∼500 ^∘^C to be ∼1.5 ky, which corresponds to a cooling rate of $∼1.7\times 10^{-9}$ ${}^{{}^{\circ}}$C s^−1^. In contrast, the laboratory cooling rate is much faster through the same temperature interval with an estimated cooling rate of $∼1.3\times 10^{-1}$ ${}^{{}^{\circ}}$C s^−1^. The significant cooling rate difference leads to a predicted ∼33% overestimate of true ancient field following the model of ref. 59 (*SI Appendix*, Fig. S3). This estimate on cooling rate effect is similar to the value of ∼30% overestimate derived from the model of ref. 61. We therefore correct our paleointensity results by a factor of 0.75. Remanence held by vortex state (pseudosingle domain) and multidomain grains is not as biased by cooling rate (62). The potential for some remanence to be held by these grains, as suggested by slightly zigzagging Arai plots (Fig. 3), could mean that this factor is an overcorrection and true paleointensity values could be slightly higher than those reported here. The cooling rate-corrected specimen paleointensity estimates together with specimen- and site-level means are shown in Fig. 4.

Anorthosite xenoliths have paleointensity results that are consistent within small regions but vary between regions. Anorthosite AX12 (28.65 ± 4.40 µT) and AX16 (33.20 ± 3.82 µT) in the Silver Bay area were emplaced ∼450 m apart and have indistinguishable paleointensity estimates (Figs. 1 and 4). Approximately 10 km to the north, anorthosite xenoliths AX11 (55.14 ± 0.05 µT) and AX13 (57.62 ± 3.20 µT) in the Kennedy Creek area were also emplaced closely (∼125 m apart) and yield similar values to one another but distinct paleointensity estimates from those of AX12 and AX16 (Figs. 1 and 4). Approximately 30 km farther to the north in the Carlton Peak region, AX6 (22.80 ± 11.41 µT), AX8 (39.40 ± 9.14 µT), and AX10 (35.20 ± 0.83 µT) are anorthosite xenoliths within 10 m of one another and also yield similar paleointensity estimates albeit with relatively large uncertainties (Fig. 4). The anorthosites of the three distinct regions captured three different intervals of geomagnetic field intensities during the emplacement and cooling of the Beaver River diabase sills ca. 1,092 Ma. To contextualize the high paleointensity values, we plot the regional mean values (*SI Appendix*, Table S3) based on specimen-level paleointensity results together with calculated paleointensity values at 22^∘^N latitude based on the paleomagnetic axial dipole moment model for the past 2 My (PADM2M) model of the time-varying geomagnetic field over the past 2 My (48) in Fig. 4*B*. The locality mean values of these three regions are plotted in Fig. 4*C* (and reported in *SI Appendix*, Table S3), where we also present the distribution of means calculated from three random values taken from the PADM2M model for a total of 10,000 iterations. In addition, we perform the same comparison with the existing paleointensity data for the Cenozoic era (the last 66 My) from the PINT database (filtered by Q${}_{PI}\geq$ 3; *SI Appendix*, Fig. S5) given that the observational dataset could record more geomagnetic field excursions or regional high-flux patches. The anorthosite xenoliths three-region mean intensity is higher than all values from the resample results of the PADM2M model and rivals the top 3% values of the resample averages from the Cenozoic data (*SI Appendix*, Fig. S5). This comparison supports that the anorthosite xenoliths record an exceptionally strong geomagnetic field ca. 1,092 Ma.

## Discussion

The crystallization of the solid inner core is an important event in the long-term evolution of Earth’s core and in sustaining the geodynamo (1). The age of the inner core in thermal evolution models relies on estimates for the thermal conductivity of iron alloys at the temperatures and pressures of the core (63). Prior to studies in the last 10 y, an accepted value of ∼30 W m^−1^ K^−1^ for this thermal conductivity constrained the timing of inner core nucleation to be during the first half of Earth’s history (8, 64). Subsequently, experimental data and ab initio simulations were interpreted to imply higher thermal conductivity values (9, 15), which in turn implied a younger age for the inner core (<700 Ma) (14). However, other experimental studies continue to indicate lower thermal conductivity values consistent with prior estimates (8, 65) with no consensus yet emerging (63, 66). These experiments are challenging to conduct and interpret given complexities such as constraining the sample thickness under high pressure and temperature conditions, the validity of applying the Wiedemann–Franz law to extrapolate thermal conductivity values based on electrical resistivity measurements (15), and propagating uncertainties from free parameters used in finite element modeling of direct thermal conduction experiments (8). Further experiments and theory are needed to explain these contrasting results which at present leave open very different trajectories for Earth’s thermal evolution. As a result, the age of the inner core is relatively unconstrained from a theoretical perspective.

The other data type that can provide insight into the long-term history of the core’s thermal regime and geodynamo is paleomagnetic data—both paleodirectional data that indicate the presence of a geomagnetic field and paleointensity data that constrain the field’s strength. Inner core nucleation would have increased the power to the geodynamo, which has the potential to manifest as an increase in Earth’s surface field (67). An approach combining dynamo simulations and theoretical scaling relationships has predicted that progressive decay of the field’s dipole moment would be followed by a rapid increase in geomagnetic field intensity soon after the onset of inner core nucleation such that a minimum in dipole moment would occur just before inner core nucleation (67). Other scenarios are possible, however, such as the model-based prediction that while power increases associated with inner core nucleation strengthen Earth’s internal magnetic field, the dynamo becomes more deeply seated in the core and diminishes the increase in magnetic field strength at Earth’s surface (68, 69). Such a scenario where the dynamo shifts to a greater depth associated with inner core nucleation led ref. 68 to conclude that the increase in power to the dynamo would be difficult to detect with paleointensity data. Ultimately, further observational paleomagnetic records are key as they hold the potential for testing different model predictions and identifying transitions in ancient field strength (22, 70).

**Fig. 6.**
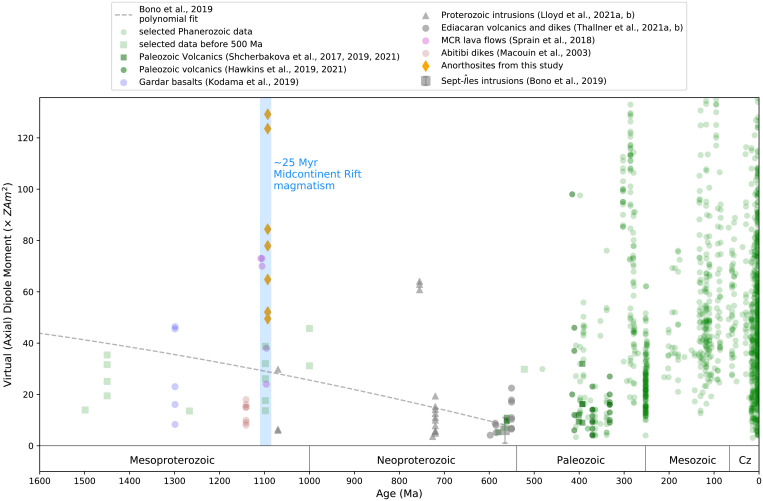
Compilation of calculated virtual (axial) dipole moment values from the PINT database (PINT v8.0.0; http://www.pintdb.org/) (71), including all Phanerozoic virtual dipole moment and virtual axial dipole moment records with Q*_PI_* values >3 and additional Neoproterozoic data from refs. 24, 72–74. Paleointensity estimates from refs. 29 and 30 are not included in the compilation due to the specimen-level double-slope behavior as discussed in the introduction section of the text. Overall, the anorthosite xenoliths from this study record a high Mesoproterozoic field exceeding the value projected by the second-order polynomial curve from ref. 22 which is based on an interpretation of there being a monotonic decay of the geodynamo through the Proterozoic. The highest site mean virtual dipole moment of the anorthosites would rank in the top 2% of those in the database for the Cenozoic Era (the last 66 My) when there was unequivocally a crystallizing inner core. The *y* axis maximum is set at the 99th percentile of the compiled Cenozoic paleointensity data.

It has been proposed that Proterozoic paleointensity data are consistent with a progressive monotonic decay leading to a minimum ca. 565 Ma in the Ediacaran period (Fig. 6) (22). This interpretation was motivated by paleointensity estimates developed from the ca. 565 Ma Sept-Îles layered mafic intrusive complex of ∼7 ZAm^2^ that are among the lowest values in the paleointensity database (Fig. 6) (22). A decay in the lead-up to this time was argued to be consistent with an absence of an inner core and a dynamo to which progressively less power was available through secular cooling (22, 67). This timing of inner core formation would favor a high core thermal conductivity (e.g., ref. 15). Paleomagnetic directional excursions (75), other weak paleointensity estimates (24), and frequent polarity reversals (76) in rocks of similar age are interpreted to be consistent with numerical simulations (77) associated with a weak dipole field.

The high paleointensity estimates from the 1.1-Gy-old Midcontinent Rift rocks challenge the hypothesized monotonic decay of the geomagnetic field strength throughout the Proterozoic Era (Fig. 6). The well-preserved ca. 1,092 Ma anorthosite xenoliths of the Beaver River diabase record a strong geomagnetic field in the late Mesoproterozoic that exceeds the modern-day field strength for which crystallization of the inner core is a power source (Figs. 4 and 6). Together with previous records obtained from the ca. 1,106 Ma Osler Volcanics of the Midcontinent Rift (31), these data indicate that appreciable power to Earth’s dynamo persisted through at least 14 Myr during the late Mesoproterozoic to maintain a strong surface field (Fig. 6). In addition to these high geomagnetic fields recorded by Midcontinent Rift rocks, the ca. 755 Ma Mundine Well dikes (73) also require a stronger geomagnetic field in the Neoproterozoic than would be predicted by a progressive Proterozoic decline. Taken together, these results call into question whether the progressively decaying polynomial fit implemented by ref. 22 is an accurate representation for the evolution of the Proterozoic geomagnetic field (Fig. 6).

The hypothesis that a weak Ediacaran geomagnetic field is a telltale sign of the lack of an inner core with core nucleation following shortly thereafter may predict that it is the most significant weak to strong field transition in the paleointensity record. However, Fig. 6 shows that transitions from low to high field intensities occurred before, during, and after the Ediacaran period. In the Ediacaran record developed to date, there is a twofold increase in Earth’s virtual dipole moment when comparing estimates from the ca. 565 Ma Sept-Îles intrusions (22) to those from ca. 550 Ma volcanics of the Skinner Cove Formation (Fig. 6) (74). In the late Mesoproterozoic, there is at least a sixfold increase within a period of ∼35 Myr from a low average virtual dipole moment of ∼13 ZAm^2^ recorded by the ca. 1,140 Ma Abitibi dikes which yielded straight Arai plots (78), to a high moment of ∼70 ZAm^2^ recorded by the ca. 1,106 Ma Osler Volcanics, with even stronger values from ca. 1,092 Ma by Beaver River anorthosite xenoliths that record virtual dipole moments up to ∼129 ZAm^2^. While the ca. 1,140 Ma Abitibi dikes’ paleointensity estimates are not as low as those of the ca. 565 Ma Sept-Îles intrusions, this virtual dipole moment increase in the Mesoproterozoic from the ca. 1,140 Ma data to the ca. 1,100 Ma data are the largest yet documented in the Precambrian on a tens of millions of years timescale (Fig. 6). The tempo and scale of this field intensity transition could match with model-based predictions associated with the onset of inner core nucleation (67). This ca. 1.1 Ga timing would be broadly consistent with the ca. 1.3 Ga onset proposed by ref. 70 albeit later given the exclusion of previous overestimated paleointensity values from the ca. 1.3 Ga Gardar basalts that are superseded by data in ref. 79. However, a model prediction of sustained strong field values following inner core nucleation is challenged by data from the ca. 1,070 Ma Bangemall Sills which include a sill with a low virtual dipole moment of ∼6.4 ZAm^2^ (Fig. 6) (73). Following the Ediacaran, there are also low paleointensity estimates from Devonian rocks such as the ca. 370 Ma dikes and lavas of the Siberian Viluy Traps that give virtual dipole moment estimates of 4.3 to 14.9 ZAm^2^ (Fig. 6) (80). These low values as well as data from the ca. 414 Ma Strathmore lava flows (27), the ca. 410 to 380 Ma lava flows of Siberia and the Kola Peninsula (25), the ca. 408 to 393 Ma Buribay volcanics (26), and the ca. 332 Ma Kinghorn volcanics (27) have led to the proposal of this interval being the Mid-Paleozoic Dipole Low (27). This Mid-Paleozoic Dipole Low is followed by high paleointensity values such that there is a sixfold increase from virtual dipole moments of ∼16 ZAm^2^ ca. 332 Ma to ∼99 ZAm^2^ ca. 308 Ma in the late Carboniferous (Fig. 6) (27).

Given that the multiple records of a weak field in the Proterozoic and Paleozoic cannot all be the minimum prior to the singular event of the onset of inner core nucleation, what processes could lead to a weak dipole at Earth’s surface even in the presence of a crystallizing inner core? Numerical models have shown that the dipole moment is sensitive to both the magnitude and spatial pattern of heat flow across the core–mantle boundary when there are strong available power sources to the geodynamo (81, 82). In such models, relatively low total heat flux across the core–mantle boundary can prevent the axial dipole from reversing, whereas a high heat flux through the boundary can result in an increase in reversal frequency and decrease in dipole intensity. The Mid-Paleozoic Dipole Low has been hypothesized to be the result of such elevated core–mantle boundary heat flux conditions at a time when there was also available power from a crystallizing inner core (80), thereby also explaining the observed low paleointensities which include values as weak as those of the ca. 565 Ma Sept-Îles intrusions (Fig. 6) (22). Mantle convection can modulate core mantle boundary heat flow through changes in the structure of the deep mantle associated with upwelling plumes (83, 84) and subducted slabs (85–87). Strong evidence for differential plate tectonic motion extends back to ca. 2.2 Ga in the Paleoproterozoic (88, 89) and potentially back to ca. 3.2 Ga in the Archean (5). Plate tectonic modulations of core mantle boundary heat flow are therefore expected throughout the Proterozoic. Such changes may explain large variability in Proterozoic paleointensity values similar to those seen in the Phanerozoic (72) and may challenge our ability to detect the increase in surface geomagnetic field strength predicted to have happened at the onset of inner core crystallization.

Overall, the high-fidelity paleointensity recorders of the Beaver River anorthosite xenoliths in the well-preserved Midcontinent Rift record strong field strengths ca. 1.1 Ga. The highest site-level value of the virtual dipole moment would rank in the top 2% of those in the database for the Cenozoic Era when there was unequivocally a crystallizing inner core. These high surface field strengths necessitate that appreciable power was provided to the late Mesoproterozoic geodynamo.

## Materials and Methods

### Sample Collection and Paleomagnetic Directions.

We collected paleomagnetic cores that are 2.5 cm in diameter along the southern and eastern Beaver Bay Complex with a particular focus on acquiring paired sites of anorthosite xenoliths and their local diabase hosts during summer field seasons in 2019 and 2020. Sample cores were collected using a handheld gasoline-powered drill and were oriented using a magnetic compass as well as a sun compass when possible. Sun compass orientations were preferentially used for determining the sample azimuth. Sister specimens underwent stepwise AF or thermal demagnetization at the University of California (UC), Berkeley Paleomagnetism Lab to isolate paleomagnetic directions (data presented in ref. 38). Based on the anorthosite thermal demagnetization results, we selected sites whose unblocking temperature ranges are narrow and near 580 ^∘^C for paleointensity experiments. Beaver River diabase sites with minimal secondary remanence were also selected for paleointensity experiments.

### Paleointensity Experiment.

A total of 86 specimens from 14 anorthosite xenoliths and a total of 69 specimens from 7 diabase sites underwent paleointensity experiments that followed the stepwise double-heating Thellier method (90) using the IZZI protocol (33) with heating steps up to 585 ^∘^C. pTRM checks were performed systematically throughout the experiment to test whether there was significant mineralogical alteration due to heating and were assessed using the SCAT parameter of ref. 46. On top of the IZZI–Thellier experiment protocol, we also performed a comparative study where we added an extra step of 20 mT AF cleaning on some of the specimens after each in-field step. The purpose was to study whether the AF cleaning could help improve experiment success rate by removing the remanence component carried by materials such as multidomain (MD) grains that contribute to nonideal paleointensity behaviors. The results were similar when this step was applied without an observed change in experimental success rate. All remanence measurements were made on a 2G Enterprises DC-SQUID superconducting rock magnetometer equipped with an automated sample changer system at the UC Berkeley Paleomagnetism Lab. The magnetometer is housed inside a three-layer magnetostatic shield that maintains background fields of less than 500 nT. Heating steps were performed using an ASC TD-48SC thermal demagnetizer with a controlled field coil that allows for a magnetic field to be generated in the oven in conjunction with a DC power supply. The thermal demagnetizer was degaussed with an AF in the axial orientation following each in-field step such that residual fields within the oven were <10 nT during zero-field steps. Samples were placed in the same location within the thermal demagnetizer for each heating step and were maintained in the same orientation with regard to the applied field. During each heating step, the oven remained at peak temperatures for 20 min to make sure each specimen reached the target temperature. An applied laboratory field of 30 µT was used for all in-field steps. All heating steps were performed in air. The temperature increments for the experiments were chosen to isolate magnetizations held by (titano)magnetite informed by the previous demagnetization data, with smaller increments performed close to ∼580 ^∘^C.

### Paleointensity Result Selection.

The following criteria were used as quality filters on the paleointensity results: 1) a maximum angular deviation (MAD) (91) of <10^∘^, 2) scatter parameter (*β*) (92) values of <15%, 3) a DANG (93) of <5^∘^, 4) fraction of remanence fitted for paleointensity estimate [FRAC (46)] >0.6, 5) scatter statistic (SCAT) (4 6) = TRUE, 6) a maximum magnetic moment difference between adjacent zero-field steps (GAP-Max) (4 6) <0.25, 7) number of pTRM checks >2, and 8) number of measurements used for paleointensity determination ≥4. MAD measures the scatter about the best-fit line through the NRM steps in the selected interval for which the intensity is defined. DANG, the deviation angle, is the angle between the best-fit direction that is free floating and the direction between the center of mass of the data and the origin of the vector component diagram (93). Both MAD and DANG assess the directional variation of the NRM, with MAD measuring the scatter in the NRM directions and DANG assessing whether the component is trending toward the origin of the Zijderveld plot. *β* is the scatter parameter of ref. 92 and is the ratio of the SE of the slope of the best-fit line of the selected NRM and pTRM points on an NRM/TRM plot to the absolute value of the slope. FRAC is the fraction of the NRM that is used in the best-fit line (46). The FRAC value was chosen to preferentially select samples with dominantly single-slope NRM/TRM plots. GAP-Max is the maximum gap between two points on the NRM/TRM plot determined by vector arithmetic. SCAT is a Boolean operator which uses the error on the best-fit slope of the selected data on the NRM/TRM plot to determine if the data are overly scattered. The parameter is used to assess pTRM checks in addition to assessing the degree to which IZZI steps are zigzagged. *β*, FRAC, GAP-Max, and SCAT are all statistics to assess the behavior of NRM/TRM plots. See the standard paleointensity definitions (94) (https://earthref.org/PmagPy/SPD/home.html) for more details. Data analysis was conducted using Thellier GUI (46) within the PmagPy software package (47).

### Rock Magnetic Experiments.

We conducted rock magnetic experiments to characterize the magnetic mineralogy and gain insights into the paleointensity results of the anorthosite and diabase. Back-field curves were measured at room temperature using a Micromag Princeton Measurements vibrating sample magnetometer (VSM) and a Lake Shore 8600 series VSM at the Institute for Rock Magnetism. Specimen MDFs were calculated based on the back-field experiments. The calculated coercivity spectra were subsequently decomposed into one or more components using skew-normal distributions following the method of ref. 55, examples of which are shown in Fig. 5. We also used an MPMS at the Institute for Rock Magnetism to aid the identification of magnetic minerals. In the field-cooled experiments, specimen magnetizations were measured upon warming following the specimen having cooled in an applied field of 2.5 T from 300 to 10 K. In the zero-field-cooled experiment, a low-temperature saturation IRM (LTSIRM) of 2.5 T was applied at 10 K after the specimen cooled in a (near-)zero field. In the room-temperature saturation IRM (RTSIRM) experiment, the sample was pulsed with a 2.5 T field at room temperature (∼300 K) and then cooled to 10 K and warmed back to room temperature in a (near-)zero field. The magnetic moment transitions at critical temperatures shown through MPMS experiments were used to identify magnetic minerals such as magnetite within specimens (53).

To further identify the magnetic carriers within the Beaver River anorthosite xenoliths and compare them with the anorthosites of the Duluth Complex Anorthositic Series rocks, we used the QDM at the UC Berkeley Paleomagnetism Lab to image a thin section of sample MS99033 from anorthosite xenolith AX16 (which yielded a ^206^ Pb/^238^ U zircon date of 1,091.83 ± 0.21 Ma) (38) and a thin section of a Duluth Complex anorthosite (Fig. 2). We use the QDM to image the magnetic field over the polished thin section surfaces with a sample–sensor distance of ∼5 µm in projective magnetic microscopy mode with a spatial resolution of 4.7 m per pixel and an instantaneous 0.9 mT bias field that is canceled during the course of measurement (95).

## Supplementary Material

Supplementary File

## Data Availability

Datasets and data analysis code are available within the GitHub repository (https://github.com/Swanson-Hysell-Group/AX_BD_PINT) that has been archived on Zenodo (https://doi.org/10.5281/zenodo.6658064) (96). The paleomagnetic data are available in the Magnetics Information Consortium (MagIC) database (https://doi.org/10.7288/V4/MAGIC/19462) (97).
