# Supplementary material for: High geomagnetic field intensity recorded by anorthosite xenoliths requires a strongly powered late Mesoproterozoic geodynamo
Source: Proc Natl Acad Sci U S A. 2022 Jul 11;119(29):e2202875119. doi: 10.1073/pnas.2202875119 (PMC9304012; doi:10.1073/pnas.2202875119)
Supplement: Supplementary File [file pnas.2202875119.sapp.pdf]

Supplementary Information for

High geomagnetic field intensity recorded by anorthosite xenoliths requires a vigorous late Mesoproterozoic geodynamo

Yiming Zhang, Nicholas L. Swanson-Hysell, Margaret S. Avery, Roger R. Fu

Yiming Zhang
E-mail: yimingzhang@berkeley.edu

This PDF file includes:

Figs. S1 to S6
Tables S1 to S3
SI References

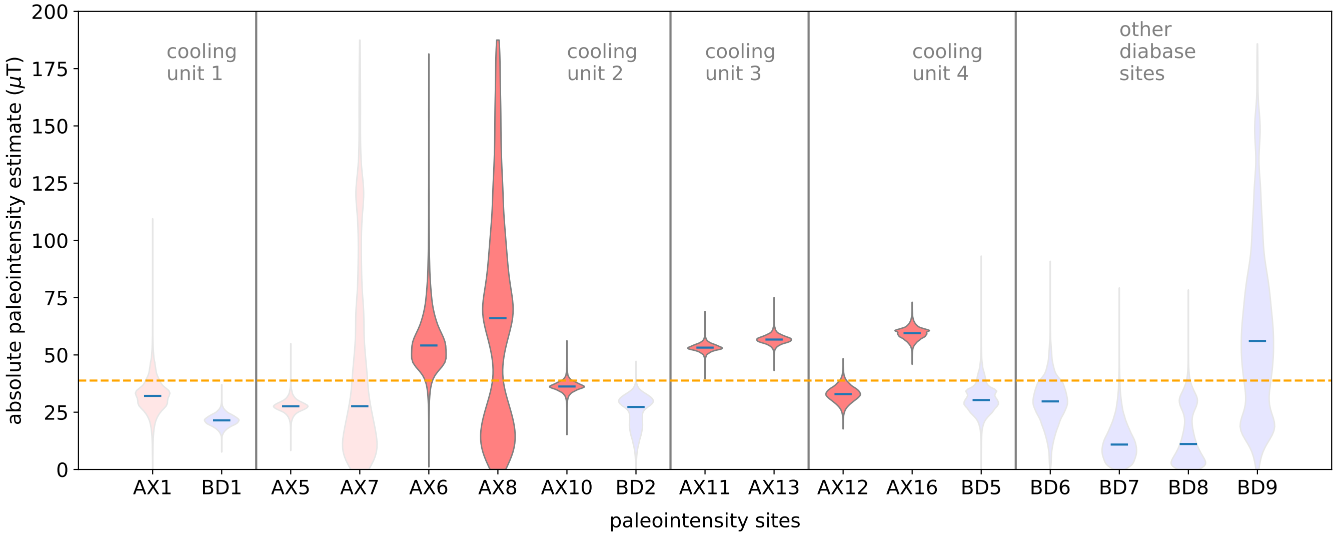

Fig. S1. Violin plots of site-level posterior paleointensity distributions estimated using the bias corrected estimation of paleointensity (BiCEP) method developed by ref. 1. Blue bars of each violin plot represent the median of the distributions. The yellow dashed line is the mean paleointensity of the posterior estimates across all sites estimated using BiCEP (38.82 $\pm$ 4.60 mT). Assuming that paleointensity estimates from specimens that come from a same cooling unit are distributed around a true paleointensity value with the various deflections being expressed as the curvature parameter of the NRM/TRM plot (2, 3), the method uses all paleointensity measurement-level data without applying selection criteria. For comparison of results from this independent method with those based on our selection (as show in Fig. 4 in manuscript), we highlight the anorthosite sites that pass our paleointensity selection and make other anorthosite and diabase more transparent. The results from the BiCEP method address the uncertainties associated with anorthosite AX6 and AX8. This is associated with the relatively variable specimen behaviors within these two sites. For sites AX10, AX11, AX13, AX12, and AX16, the posterior probability distributions have very narrow bounds, consistent with the interpretation that these anorthosites are faithful paleointensity recorders that have high-quality paleointensity behaviors. AX–anorthosite xenolith site; BD–Beaver River diabase site. The grouping of cooling units is based on the spatial proximity between site locations and the inclusion relationship of host diabase and anorthosite xenoliths reported by ref. 4.

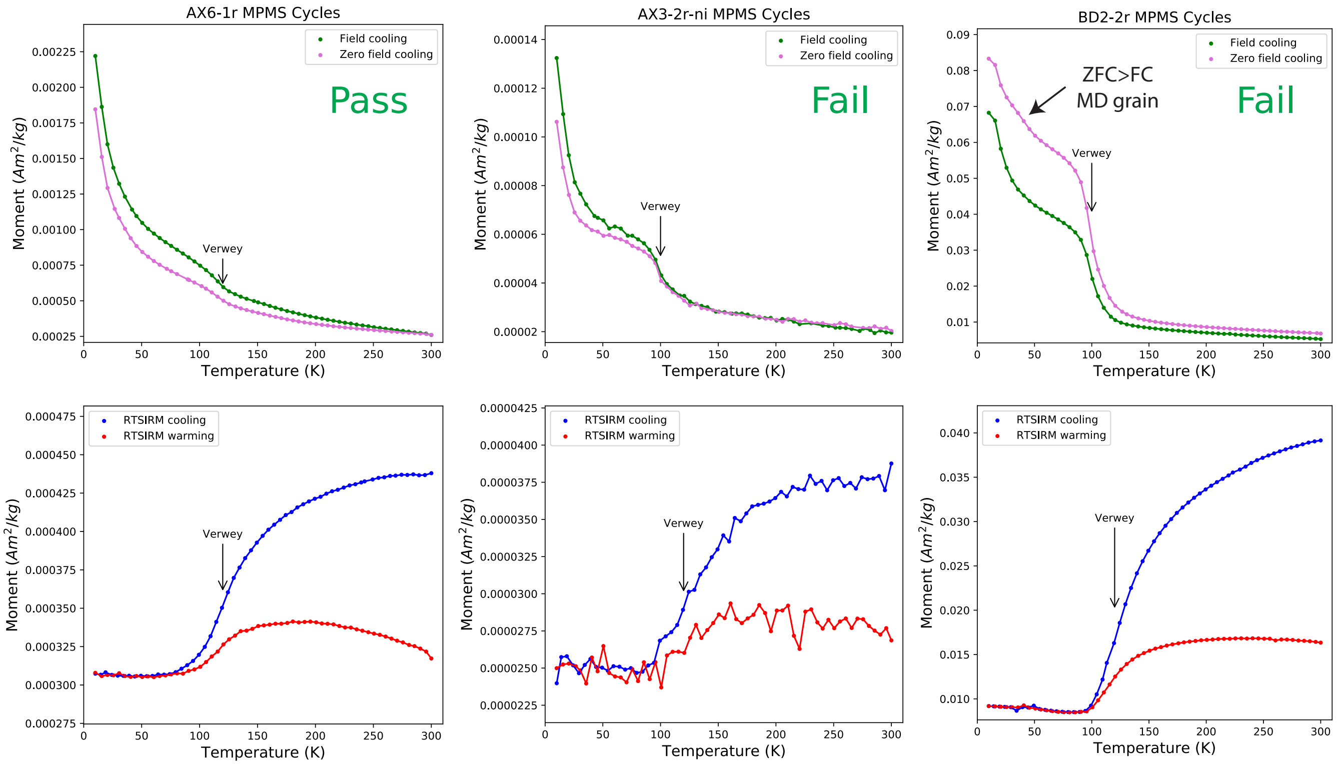

Fig. S2. Low-temperature magnetic property measurement system (MPMS) experiment results. "Pass" or "Fail" represents whether their sister specimens passed our paleointensity selection or not. In the field-cooled (FC) experiments, the magnetization was measured upon warming following the specimen having cooled in an applied field of 2.5 T from 300 to 10 K. In the zero-field-cooled (ZFC) experiment, a low-temperature saturation isothermal remanence (LTSIRM) of 2.5 T was applied at 10 K after the specimen cooled in a (near-)zero field. In the room-temperature saturation isothermal remanence (RTSIRM) experiment, the sample was pulsed with a 2.5 T field at room temperature ($\sim$300 K) and then cooled to 10 K and warmed back to room temperature in a (near-) zero field. Specimen AX6-1r is from anorthosite AX6 which passed our paleointensity selection. It has a well-defined Verwey transition $\sim$120 K (5). Specimens AX3-2r-ni and BD2-2r show Verwey transition but the transition temperatures are suppressed below $\sim$120 K. Specimen BD2-2r has a consistently higher moment during the zero-field-cooled step than during the field-cooled step. This is consistent with the interpretation that multidomain (MD) magnetic carriers exist in significant quantity in this specimen (6).

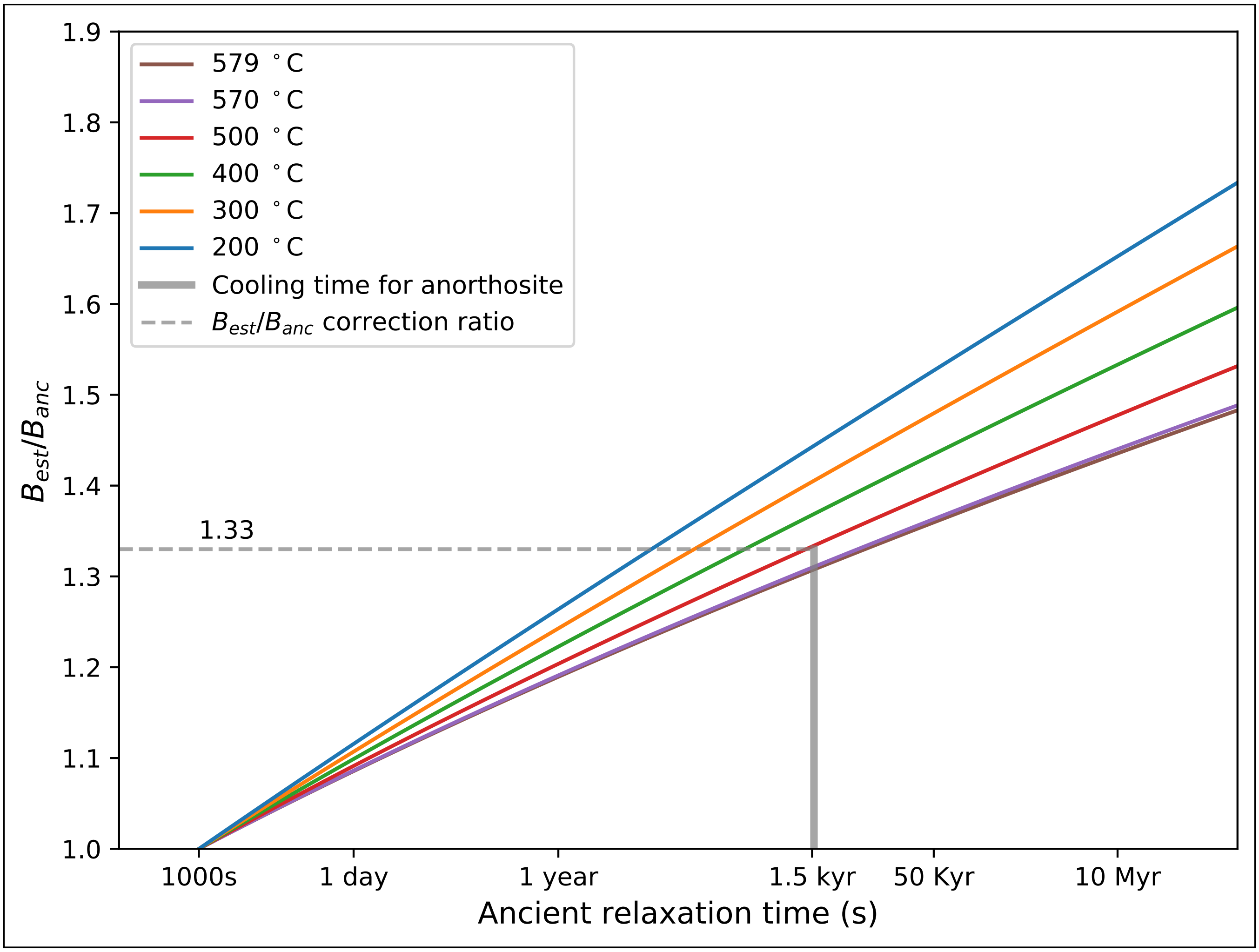

Fig. S3. Graph of predicted paleointensity overestimate due to slow cooling of the intrusive Beaver River diabase and anorthosite xenoliths relative to the cooling rate in laboratory following the model of ref. 7. Because the majority of the anorthosites have unblocking temperatures between 500℃ and 580℃, we estimate that the slow cooling during natural remanence acquisition could have resulted in a 33% overestimate. Thus, a correction factor of 0.75 is applied at specimen level for the paleointensity summary plot.

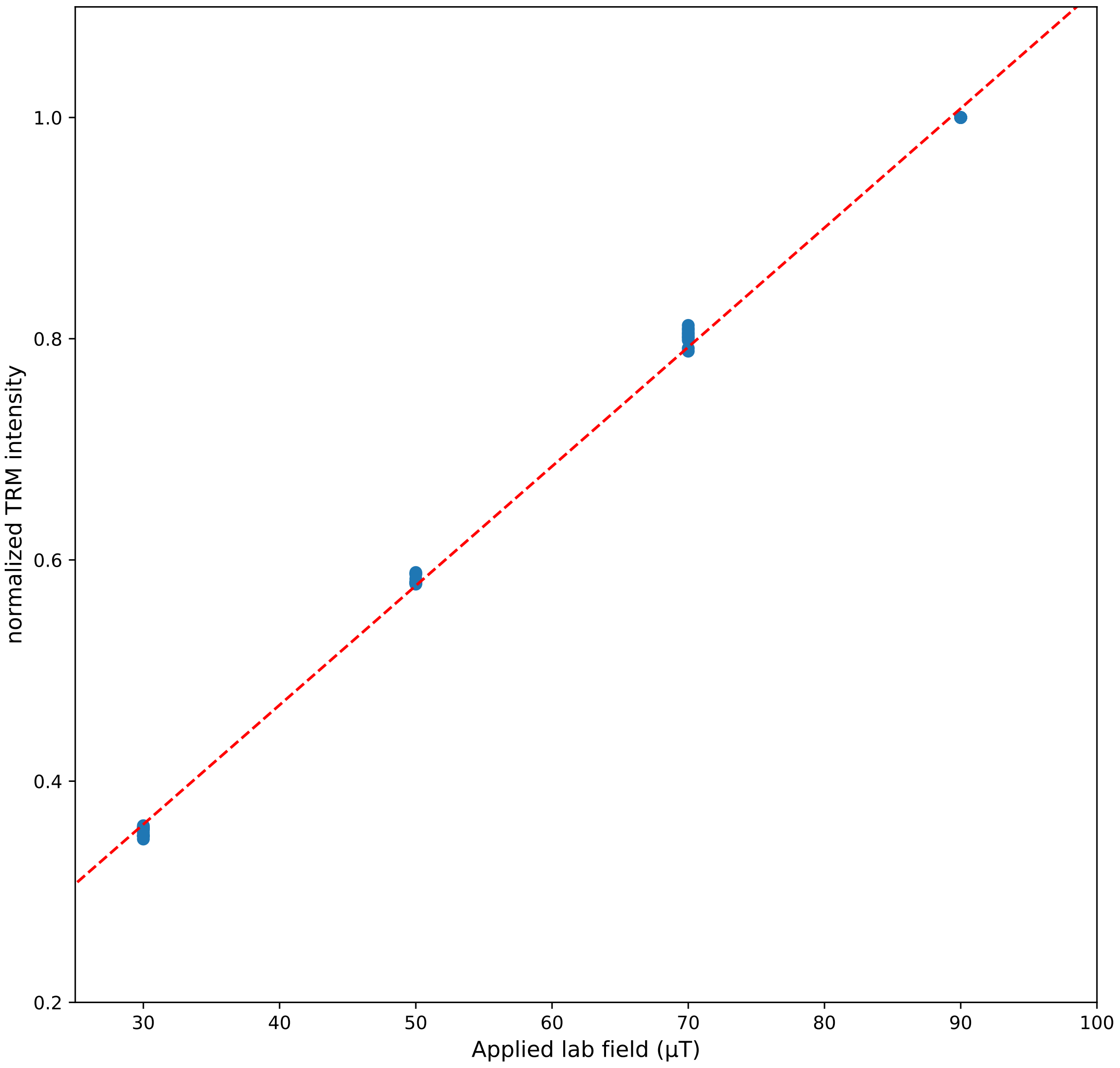

Fig. S4. Plot of linear thermal remanent magnetization acquisition experiment results. After IZZI-type Thellier paleointensity experiments, full TRMs were imparted on the same specimens in known lab fields of 30, 50, 70, and 90 μT. The red dashed line shows a linear fit through the data points. The results show that the anorthosite xenoliths do not acquire saturation remanence or display non-linear remanence acquisition under fields relevant to this study. Therefore, non-linear acquisition correction is not needed for our paleointensity results.

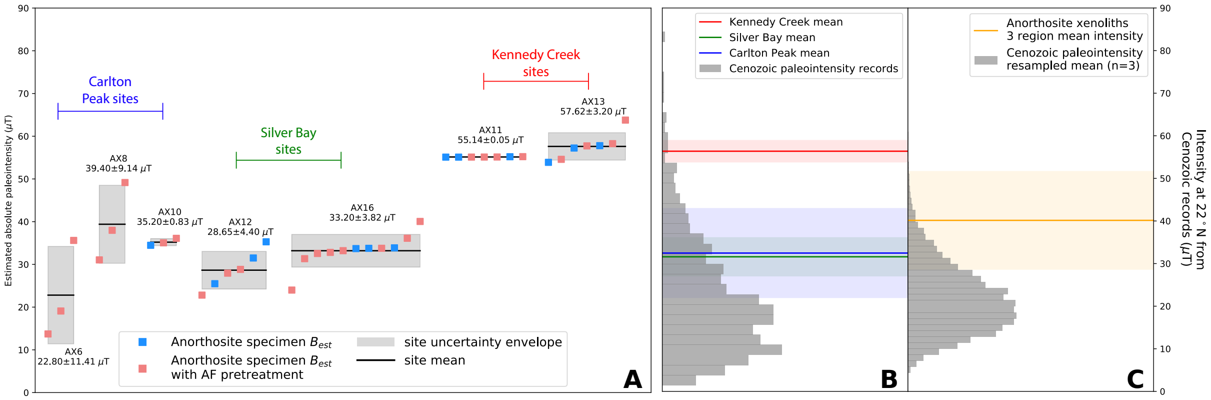

Fig. S5. Data from this study as in Figure 4 of the main text, but compared to the Cenozoic paleointensity database rather than the PADM2M model. A) Summary plot of individual specimen absolute paleointensity results (square symbols) and their averages and standard deviations at site level (black bars with grey uncertainty boxes) from this study. All results are corrected for cooling rate with a factor of 0.75. Each 'AX' site is an individual anorthosite xenolith within the Beaver River diabase. The sites with successful experiments come from 3 regions which would have cooled at distinct times yielding similar estimates within the each region with differences between regions. B) Specimen level means calculated for these regions are compared to the distribution of intensities calculated from the existing paleointensity data in the Cenozoic (past 66 Myr) in the PINT database (PINT v8.0.0; http://www.pintdb.org/; 8) at the latitude corresponding to the paleolatitude of study region (22°N). C) The mean of the 3 regional means is compared to means calculated from 3 random values drawn from the Cenozoic data. The paleointensity records used for resampling are filtered for $Q_{PI} \geq 3$ (9). The distribution represents a total of 10,000 iterations of taking 3 random draws and calculating the mean.

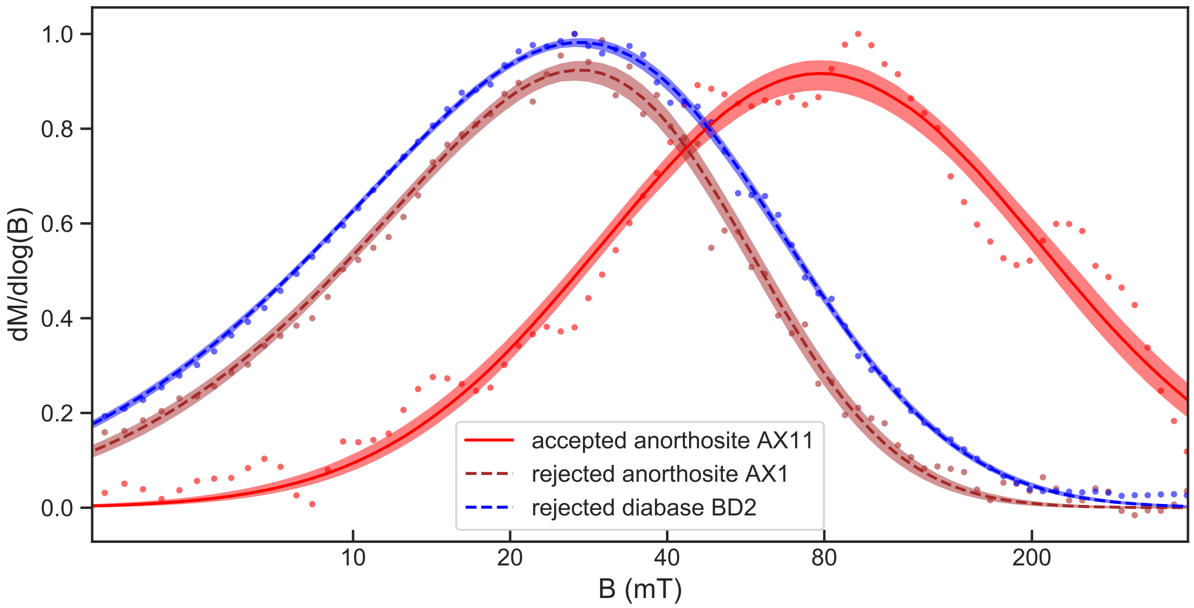

Fig. S6. Example coercivity spectra of anorthosite and diabase specimens from sites that pass or fail our paleointensity selection criteria. The specimens and fits are the same as those shown in Figure 5 in the manuscript. Data points used for fitting the coercivity curves are shown in this figure.

Table S1. Specimen paleointensity results that passed our selection. B_{anc} is the calculated ancient field intensity over the chosen temperature interval in μT. T_{min} and T_{max} indicate the temperature interval over which the best fit for paleointensity was defined. N is the number of steps used within the selected interval for paleointensity determination. FRAC is the fraction of remanence used for fitting. NpTRM shows the number of pTRM checks within the selected interval for paleointensity determination. β is the scatter parameter. GAP-MAX is the maximum magnetization gap between two adjacent steps. MAD is the maximum angle of deviation. DANG is the deviation angle. SCAT is the scatter parameter. inc_{tc} is the tilt-corrected inclination. Paleolatitude is calculated from the inclination values reported in (4). γ is the gamma statistic that measures the angle between the last pTRM step used for paleointensity determination and the applied field direction. V(A)DM is the virtual (axial) dipole moment reported in 10^{21} Am^2 (ZAm^2).

Site	Specimen	B_{anc}	T_{min}	T_{max}	N	FRAC	NpTRM	β	GAP-MAX	MAD (°)	DANG (°)	SCAT	inc_{tc}	Paleolatitude	γ	VDM (ZAm^2)
AX6	AX6-2a	13.72	400	585	18	0.70	10	0.04	0.12	3.4	3.4	PASS	38.9	22.0	2.7	29.8
AX6	AX6-3a	19.09	400	585	18	0.76	10	0.04	0.10	4.3	2.9	PASS	38.9	22.0	3.2	41.4
AX6	AX6-1a	35.61	475	585	15	0.60	10	0.02	0.16	2.9	1.7	PASS	38.9	22.0	2.0	77.3
AX8	AX8-3a	31.05	400	580	17	0.75	9	0.03	0.14	4.4	2.2	PASS	40.3	23.0	11.2	66.5
AX8	AX8-2a	37.98	400	580	17	0.63	9	0.04	0.16	3.2	1.3	PASS	40.3	23.0	3.7	81.4
AX8	AX8-1a	49.16	425	566	13	0.60	8	0.07	0.20	5.3	3.0	PASS	40.3	23.0	7.0	105.3
AX10	AX10-1a	34.47	425	585	17	0.78	10	0.06	0.24	5.6	2.6	PASS	36.6	20.4	4.7	76.3
AX10	AX10-2a	35.05	450	585	16	0.62	10	0.04	0.20	5.3	2.2	PASS	36.6	20.4	7.8	77.6
AX10	AX10-3a	36.10	425	585	17	0.69	10	0.04	0.24	4.0	1.6	PASS	36.6	20.4	5.8	79.9
AX11	AX11-1a	55.11	425	560	10	0.67	6	0.08	0.21	5.3	2.5	PASS	35.2	19.4	5.9	123.5
AX11	AX11-2a	55.11	400	560	11	0.65	6	0.07	0.21	3.9	1.5	PASS	35.2	19.4	9.9	123.5
AX11	AX11-4a	55.12	500	570	11	0.66	8	0.09	0.20	1.5	3.3	PASS	35.2	19.4	5.6	123.5
AX11	AX11-6a	55.13	200	562	14	0.69	7	0.07	0.17	4.0	1.3	PASS	35.2	19.4	3.0	123.5
AX11	AX11-9a	55.14	100	562	15	0.67	7	0.06	0.17	5.3	1.1	PASS	35.2	19.4	11.9	123.5
AX11	AX11-3a	55.20	400	560	11	0.66	6	0.08	0.21	4.0	2.1	PASS	35.2	19.4	8.2	123.7
AX11	AX11-5a	55.22	400	566	14	0.73	8	0.05	0.18	2.9	0.9	PASS	35.2	19.4	1.9	123.7
AX12	AX12-14a	22.81	475	585	16	0.75	10	0.06	0.17	1.7	0.6	PASS	55.2	35.7	0.9	41.5
AX12	AX12-1a	25.48	0	580	21	0.97	9	0.03	0.17	5.5	4.7	PASS	55.2	35.7	10.0	46.3
AX12	AX12-6a	27.95	425	564	12	0.69	7	0.08	0.22	3.6	2.1	PASS	55.2	35.7	5.0	50.8
AX12	AX12-8a	28.83	475	565	12	0.75	8	0.06	0.25	1.5	2.3	PASS	55.2	35.7	1.6	52.4
AX12	AX12-4a	31.50	500	585	14	0.66	10	0.05	0.24	3.9	3.2	PASS	55.2	35.7	11.4	57.3
AX12	AX12-2a	35.29	425	570	14	0.70	8	0.05	0.24	3.4	2.4	PASS	55.2	35.7	7.0	64.2
AX13	AX13-3a	53.89	100	550	12	0.71	5	0.08	0.22	5.9	1.9	PASS	35.1	19.4	3.4	120.9
AX13	AX13-7a	54.59	475	585	15	0.61	10	0.03	0.20	3.6	2.1	PASS	35.1	19.4	11.6	122.4
AX13	AX13-4a	57.24	200	550	11	0.65	5	0.04	0.18	8.8	1.1	PASS	35.1	19.4	3.7	128.4
AX13	AX13-6a	57.74	200	555	12	0.70	6	0.06	0.22	3.8	1.5	PASS	35.1	19.4	4.5	129.5
AX13	AX13-2a	57.79	200	555	12	0.71	6	0.05	0.20	8.1	2.2	PASS	35.1	19.4	4.1	129.6
AX13	AX13-9a	58.26	0	564	17	0.94	7	0.03	0.16	6.0	1.1	PASS	35.1	19.4	2.6	130.7
AX13	AX13-8a	63.78	450	570	13	0.81	8	0.02	0.24	3.7	1.8	PASS	35.1	19.4	5.1	143.0
AX16	AX16-15a	23.99	475	570	13	0.61	8	0.07	0.17	3.1	1.4	PASS	49.2	30.1	2.6	46.8
AX16	AX16-13a	31.34	400	570	15	0.72	8	0.07	0.20	3.5	1.9	PASS	49.2	30.1	1.1	61.2
AX16	AX16-16a	32.56	400	562	13	0.65	7	0.09	0.18	5.1	2.9	PASS	49.2	30.1	2.0	63.6
AX16	AX16-14a	32.81	400	565	14	0.63	8	0.08	0.14	3.8	2.1	PASS	49.2	30.1	6.0	64.1
AX16	AX16-11a	33.21	450	570	14	0.70	8	0.06	0.15	2.9	2.1	PASS	49.2	30.1	4.6	64.9
AX16	AX16-4a	33.70	425	575	15	0.76	9	0.05	0.21	4.8	1.1	PASS	49.2	30.1	4.2	65.8
AX16	AX16-1a	33.76	200	564	15	0.87	7	0.06	0.22	5.6	2.4	PASS	49.2	30.1	4.5	65.9
AX16	AX16-5a	33.77	425	560	10	0.65	6	0.10	0.24	7.4	3.9	PASS	49.2	30.1	4.0	65.9
AX16	AX16-2a	33.87	425	564	12	0.65	7	0.07	0.22	4.3	1.4	PASS	49.2	30.1	4.9	66.1
AX16	AX16-9a	36.12	500	585	15	0.61	10	0.04	0.17	3.0	1.2	PASS	49.2	30.1	4.3	70.5
AX16	AX16-10a	40.05	510	585	14	0.62	10	0.04	0.20	2.8	1.1	PASS	49.2	30.1	5.5	78.2

Table S2. Summary statistics for the Q_{PI} quality criteria of (9).

Site	N	Age (Ma)	Method	AGE	STAT	TRM	ALT	MD	ACN	TECH	LITH	QPI
AX6	3	1091.8	T+	1	0	1	1	1	1	0	0	5
AX8	2	1091.8	T+	1	0	1	1	1	1	0	0	5
AX10	3	1091.8	T+	1	0	1	1	1	1	0	0	5
AX11	7	1091.8	T+	1	1	1	1	1	1	0	0	6
AX12	6	1091.8	T+	1	1	1	1	1	1	0	0	6
AX13	7	1091.8	T+	1	1	1	1	1	1	0	0	6
AX16	11	1091.8	T+	1	1	1	1	1	1	0	0	6

Table S3. **Summary paleointensity result statistics at site-level, region-level, and overall arithmetic means. The statistics names are the same as in Table S1. n represents the total number of specimen- or site- or region-level results used for calculating mean values. inc_{tc} is the tilt-corrected mean inclination. Site-level means for AX6, AX8, AX10, AX11, AX12, AX13, AX16 are calculated based on specimen results. Regional means for Carlton Peak, Kennedy Creek, and Silver Bay are calculated by grouping specimen results from AX6 and AX8 and AX10, AX11 and AX13, AX12 and AX16, respectively. Overall site mean values are calculated using all site-level results in this table. Overall region-mean values are calculated using all regional mean results in this table. All paleointensity values and corresponding virtual dipole moments are corrected for cooling rate by a factor of 0.75. Taking a threshold p value of 0.01, the independent two-sample t-test results show that specimen paleointensity distributions of site AX12 and AX16, and those of site AX11 and AX13 are indistinguishable; region Kennedy Creek have distinguishable distributions from the other two regions; Silver Bay region and Carlton Peak region values are indistinguishable.**

		B_{anc}	n	FRAC	GAP-MAX	MAD (°)	DANG (°)	inc_{tc}	Paleolatitude	γ	VDM (ZAm^2)
specimen mean for sites	AX6	22.8	3	0.69	0.13	3.6	2.7	38.9	22.0	2.6	49.5
	AX8	39.4	3	0.66	0.17	4.3	2.2	40.3	23.0	7.3	84.4
	AX10	35.2	3	0.70	0.23	5.0	2.1	36.6	20.4	6.1	77.9
	AX11	55.2	7	0.68	0.19	3.8	1.8	35.2	19.4	6.6	123.6
	AX12	28.6	6	0.75	0.22	3.3	2.5	55.2	35.7	6.0	52.1
	AX13	57.6	7	0.73	0.2	5.7	1.7	35.1	19.4	5.0	129.2
	AX16	33.2	11	0.68	0.19	4.2	2.0	49.2	30.1	4.0	64.8
specimen mean for regions	Carlton Peak	32.5	9	0.68	0.17	4.3	2.3	38.6	21.8	5.3	70.6
	Kennedy Creek	56.4	14	0.70	0.20	4.8	1.7	35.2	19.4	5.8	126.4
	Silver Bay	31.6	17	0.71	0.20	3.9	2.2	51.3	32.1	4.7	60.3
overall site mean		38.9	7								83.1
overall region mean		40.2	3								85.8

References

1. B Cych, M Morzfeld, L Tauxe, Bias corrected estimation of paleointensity (BiCEP): An improved methodology for obtaining paleointensity estimates. *Geochem. Geophys. Geosystems* **22** (2021).
2. Y Arai, Secular variation in intensity of the past geomagnetic field. *M.Sc. thesis, Univ. Tokyo, Tokyo, Jpn.* (1963).
3. GA Paterson, A simple test for the presence of multidomain behavior during paleointensity experiments. *J. Geophys. Res.* **116** (2011).
4. Y Zhang, NL Swanson-Hysell, MD Schmitz, JD Miller, MS Avery, Synchronous emplacement of the anorthosite xenolith-bearing Beaver River diabase and one of the largest lava flows on Earth. *Geochem. Geophys. Geosystems* (2021).
5. EJW Verwey, Electronic conduction of magnetite (Fe3O4) and its transition point at low temperatures. *Nature* **144**, 327–328 (1939).
6. B Carter-Stiglitz, et al., Low-temperature magnetic behavior of multidomain titanomagnetites: TM0, TM16, and TM35. *J. Geophys. Res. Solid Earth* **111** (2006).
7. SL Halgedahl, R Day, M Fuller, The effect of cooling rate on the intensity of weak-field trm in single-domain magnetite. *J. Geophys. Res. Solid Earth* **85**, 3690–3698 (1980).
8. RK Bono, et al., The PINT database: A definitive compilation of absolute palaeomagnetic intensity determinations since 4 billion years ago. *Geophys. J. Int.* (2021).
9. AJ Biggin, GA Paterson, A new set of qualitative reliability criteria to aid inferences on palaeomagnetic dipole moment variations through geological time. *Front. Earth Sci.* **2** (2014).
